# Defining tissue resident vascular stem cells

**DOI:** 10.18632/oncotarget.21389

**Published:** 2017-09-29

**Authors:** Jatin Patel, Prudence Donovan, Kiarash Khosrotehrani

**Affiliations:** Jatin Patel: UQ Diamantina Institute, The University of Queensland, Brisbane, Queensland, Australia

**Keywords:** endothelial progenitor cells, angiogenesis, vasculogenesis, endothelium, tissue regeneration

The vascular system is an essential component of tissue homeostasis as well as the response to injury, given its widespread presence and essential role in most tissues including cancer. During embryonic development, vessels form from the periphery of blood islands as separate units that end up joining in a process called vasculogenesis [1]. However, in adults, formation of new blood vessels in response to insults or physiological signals is considered to result from the extension of existing vessels, defined as angiogenesis [2]. More recently, neo-vessel formation in adults has also been associated with activity of endothelial progenitors and their ability to assemble the intimal layers of mature vessels [1-3]. This initiated the concept that an endothelial progenitor with capacity to self-renew and form blood vessels, not only existed in adult tissues but could be transferred to a new environment to form neo-vessels, potentially recapitulating some features of vasculogenesis. However, the definition, cell lineage and possible hierarchy of such precursors remained controversial [3-5].

The endothelial cells from a variety of vascular beds have been shown to be hetergogeneous in terms of their levels of cell surface expression or in terms of their proliferation and self-renewal capacity [5, 6]. Yoder et al have elegantly demonstrated this through the culture of human endothelial colony forming cells (ECFC) from umbilical cord blood [5]. In a recent publication, we have now delineated this further by demonstrating the endothelial hierarchy as three distinct populations via *in vivo* modelling systems. We termed the populations as an endovascular progenitor (EVP) (CD31-/loVEGFR2lo/intracellular), transit amplifying (TA) (CD31intVEGFR2lo/intracellular) and definitive differentiated (D) (CD31hiVEGFR2hi/extracellular) endothelial cells. In the temporally-defined context of wound healing, these three populations appeared sequentially in quantities suggestive of a hierarchy, as confirmed by lineage tracing using the *Cdh5-Cre*^ERt2^*/ROSA-YFP*. Inherent stem/progenitor capacity of the EVP was confirmed through transplantation experiments, whereby EVP isolated from a tumor setting of a donor were implanted in a Matrigel plug of a host. In this context EVP had a capacity to form neovessels in the host, whereas TA and D did not display this capacity. Furthermore, EVP but not TA and D cells had an *in vitro* colony forming capacity when isolated from either an active wound healing, tumor or the normal abdominal aorta environment.

It has been questioned whether bone marrow was the primary source of these endothelial progenitors during tissue repair following the generation of GFP+ bone marrow chimeras. The wounded GFP+ chimeras were devoid of any donor derived newly formed vessels within the wound granulation tissues [6]. This excluded any significant bone marrow or hematopoietic contribution to neo-vessel formation in this context. Okuno et al, also confirmed our findings by demonstrating that bone-marrow derived cells mainly contribute to vessel formation with skin wounds through paracrine activity [7].

A major roadblock in defining *in vivo* tissue resident endothelial progenitors has been determining their location along the vascular structure. Although here the precise location of EVPs remains to be determined within tissue resident structures, there is evidence to suggest that these cells lie in existing vessels in the tissue surrounding the wound or the tumor. To address this question we used the vascular specific *Cdh5-Cre*^ERt2^*/ ROSA-YFP*, a gold standard mouse model in tracing vascular populations. Using this model, we demonstrated through flow cytometry and immunofluorescence the migration of EVP cells from vascular beds into the center of the granulation tissue of Day 0 wounds.

Gene expression analysis of endothelial progenitors to differentiated cells in mice *in vivo* [6] and human cells *in vitro* [8], demonstrated strong expression of key markers classically defining endothelial cells and their function in differentiated cells. Most surprising was the identification of genes that delineated the endothelial progenitor populations with striking similarities between human and mouse populations. Many genes upregulated here were related to growth factor signaling, such as those from the *pdgf* and *egf* families. Both these pathways are potent activators in cell migration and maintenance of stem cell populations. For the first time, a distinct picture of the gene profile for *in vivo* endothelial progenitors has been described. This is a significant stride forward in defining the molecular drivers of endothelial progenitors and understanding their role in vascular homeostasis and/or pathologies.

Therefore, our work as well as others propose a new definition of endothelial progenitors, a novel population of vessel resident EVPs participating in pathological and physiological neovessel formation. Their molecular definition and sequential differentiation forming immature and then definitive vessels provides important insights in the activity of endothelial progenitors *in vivo*, in a variety of vascular beds including tumour vessels. This paradigm shift in our understanding of vascular resident endothelial progenitors in tissue regeneration opens new avenues for better understanding of vascular biology with potential applications in oncology.
